# Mucoepidermoid carcinoma (MEC) and adenosquamous carcinoma (ASC), the same or different entities?

**DOI:** 10.1038/s41379-022-01100-z

**Published:** 2022-07-23

**Authors:** Valerie A. White, Martin D. Hyrcza, Jochen K. Lennerz, Julia Thierauf, Dilani Lokuhetty, Ian A. Cree, Blanca Iciar Indave

**Affiliations:** 1 International Agency for Research on Cancer, Lyon, France; 2 Department of Pathology and Laboratory Medicine, University of Calgary, Arnie Charbonneau Cancer Institute, Calgary, AB Canada; 3 Department of Pathology, Center for Integrated Diagnostics, Massachusetts General Hospital/Harvard Medical School, Boston, MA USA; 4 Department of Pathology, University of Colombo, Colombo, Sri Lanka

**Keywords:** Diagnostic markers, Tumour biomarkers, Head and neck cancer

## Abstract

Mucoepidermoid carcinoma (MEC) and adenosquamous carcinoma (ASC) have overlapping histopathological appearances and sites of occurrence, which may cause diagnostic difficulty impacting subsequent treatment. We conducted a systematic review of the scientific literature to determine whether molecular alterations were sufficiently different in MEC and ASC to aid in classifying the two entities. We searched Medline, Embase and Web of Science for studies reporting molecular determinations of ASC and/or MEC and screened retrieved records for eligibility. Two independent researchers reviewed included studies, assessed methodological quality and extracted data. Of 8623 identified records, 128 articles were included for analysis: 5 which compared the two tumors in the same investigation using the same methods and 123 which examined the tumors separately. All articles, except one were case series of moderate to poor methodological quality. The 5 publications examining both tumors showed that 52/88 (59%) MEC and 0% of 110 ASC had rearrangement of the *MAML2* gene as detected by FISH and/or RT-PCR, but did not investigate other genes. In the entire series MEC had *MAML2* gene rearrangement in 1337/2009 (66.6%) of tumors studied. The articles examining tumors separately found that MEC had mutations in *EGFR* (11/329 cases, 3.3%), *KRAS* (11/266, 4.1%) and *ERBB2* (9/126, 7.1%) compared with ASC that had mutations in *EGFR* (660/1705, 38.7%), *KRAS* (143/625, 22.9%) and *ERBB2* (6/196, 3.1%). The highest level of recurrent mutations was in pancreatic ASC where (108/126, 85.7%) reported mutations in *KRAS*. The *EGFR* mutations in ASC were similar in number and kind to those in lung adenocarcinoma. By standards of systematic review methodology and despite the large number of retrieved studies, we did not find adequate evidence for a distinctive molecular profile of either MEC or ASC that could definitively aid in its classification, especially in histologically difficult cases that are negative for *MAML2* rearrangement. The case series included in this review indicate the relevance of *MAML2* rearrangement to support the diagnosis of MEC, findings that should be confirmed by additional research with adequate study design.

## Introduction/background

The correct classification of a tumor is an essential step in diagnosis and forms the cornerstone of an individual cancer patient’s clinical care and outcome. Tumors are classified by pathologists on microscopic examination of the tumor architecture and cytology, as well as ancillary techniques, such as histochemical and immunohistochemical stains, cytogenetics, and molecular studies^1^. Molecular techniques have increasingly influenced tumor classification, allowing further classification of tumors with similar histopathological appearances and immunohistochemical staining patterns.

In certain pathology subspecialties, such as hematopathology, neuropathology, and soft tissue pathology, precise and detailed tumor diagnosis is frequently based on the molecular alterations of particular tumor types. In other areas of pathology there are ongoing discussions on whether tumor types should be defined primarily based on histopathology, on their molecular alterations^2–4^, or on other characteristics. The difficulties are in part due to the presence of multiple different and frequently exclusive alterations in histopathologically identical tumors for which the diagnostic and clinical relevance is not always clear. On the other hand, specific molecular alterations do not always segregate closely with known neoplasms and variants^5^.

The diagnostic and clinical relevance of molecular alterations has not always been clearly established and definitive evidence is often not available^6,7^. In terms of scientific evidence, the simple observation of a mutation in a number of reported cases can’t be considered as sufficient evidence of an association between the presence of the mutation and a definitive diagnosis^8,9^. The appropriate interpretation of a correlation (as opposed to an association) has been extensively discussed and applied in pathology to define biomarkers as surrogate endpoints for clinical trials, where solid scientific evidence is required by epidemiological, therapeutic, and/or pathophysiological studies along with an appropriate design and statistical analysis^10,11^. These strict scientific requirements when describing a clinical-pathological relationship also need to be applied when considering mutations for protocols in cancer diagnosis and tumor classification.

This systematic review focuses on two tumors that can have similar histopathological appearances and overlapping sites of occurrence which may cause difficulty in correct classification, and subsequent treatment in some instances. Adenosquamous carcinoma (ASC) is a biphasic epithelial neoplasm that frequently occurs in the lung, but is also documented in the pancreas, cervix, gallbladder, head and neck, and other sites^12^. As the name implies it is composed of both adenocarcinoma and squamous carcinoma components. Although the exact amount of each component has not been defined, the WHO Classification of Tumors states that there should be at least 10% of each component to make this diagnosis^1,12^ although by sampled tumor volume the glandular component may range from 10 to 40%^13^ and some publications in this review used a 30% cutoff^14^. Each component can show the growth patterns seen in each of these carcinomas when they are present alone. The definition of ASC was furthered refined by including the presence of an in-situ squamous component implying a surface origin, as well as overt keratinization^15^. Due to its rarity, the molecular properties of this neoplasm have not been well studied and a definitive molecular signature has not been established.

Mucoepidermoid carcinoma (MEC) is also a biphasic epithelial tumor that arises from the submucosa and is characterized by a mixture of mucinous (goblet) cells and an epidermoid component without overt keratinization^1,16^. It also has a third nondescript intermediate-type cell which is smaller and contains neither mucin nor abundant cytoplasm. In contrast to ASC, MEC does not show squamous pearls and often lacks intercellular bridges. There is no in-situ carcinoma in the overlying epithelium. When these tumors are low-grade, they often form large mucous-filled cysts, while higher grade tumor show more solid morphology, fewer mucinous cells, and more invasive growth. The most common location for MEC is in the salivary glands, but it also occurs in the lung and other sites^1,16^. In 1994 a recurrent translocation t(11;19)(q14-21;p12) was described by Nordkvist et al. in this neoplasm^17^. Subsequent studies showed that this translocation fused exon 1 of the *CRTC1* gene on chromosome 19p13 with exons 2–5 of the *MAML2* gene at chromosome 11q21^18^. This distinctive fusion is present in 40–90% of MEC, depending on the study^19–23^. Among the cases of salivary MEC lacking the *MAML2* gene rearrangement, some show morphology classical of MEC, while others resemble ASC. It is currently an unresolved question how to accurately separate MEC without *MAML2* rearrangement from ASC.

The objective of this systematic review was to summarize the available evidence on molecular alterations in MEC and ASC to determine if these two neoplasms share the same or different molecular pathology which could be used to help distinguish these neoplasms in histologically difficult cases. We specifically aimed to identify frequent mutations and describe the frequency with which these mutations have been reported.

## Methods

### Literature search and study selection

We conducted a systematic review to identify peer-reviewed articles reporting molecular determinations of ASC and MEC following the recommendations of the Preferred Reporting Items for Systematic Reviews and Meta-analyses (PRISMA)^24,25^. We prepared a protocol following these guidelines and registered it in the International Prospective Register of Systematic Reviews (Prospero CRD42019129352).

A search strategy was developed in collaboration with qualified librarians and tailored search strings were utilized to search Medline, Embase and Web of Science for relevant publications; the Cochrane library and PROSPERO register were also consulted. Search strings contained keywords and database-specific terms (MeSH headings, Emtree terms and exploded terms) for the three major concepts of ASC, MEC and molecular determinations. Multiple variations of search terms were combined to produce different sets of results. The full search strategy is presented in Supplementary materials (S-Table 1).

### Eligibility criteria

#### Inclusion criteria

We included studies reporting on patients with histopathologically confirmed diagnoses of MEC or ASC that were investigated by molecular genetic methods. As a first step we included studies reporting results comparing both tumors in the same investigation by the same methods. Although we did not limit studies to those that determined the presence of the specific translocation t(11;19)(q21;p13) and/or rearrangements involving the *MAML2* gene, these were the only investigations found in this group of publications. As we retrieved so few publications that compared these tumors directly, we decided to include studies reporting on mutations of three of the most frequent and potentially important genes for either of the tumor types. We considered studies using all types of molecular investigation [fluorescence in-situ hybridization (FISH), polymerase chain reaction (PCR), all types of sequencing and karyotyping] for inclusion, and included studies that utilized any type of observational or experimental study design. This included clinical trials, randomized or not, cohort, case control studies, cross sectional studies and also case series.

We included articles published in English, French, German, and Spanish between 1990 and October 7, 2021.

#### Exclusion criteria

We excluded non original research such as narrative reviews, letters to the editor and expert opinion; basic research, including animal and in vitro studies; computer modeling studies; reports from conferences or annual meetings; and reports of large series of other tumors containing ≤2 tumors of interest.

Duplicates were eliminated and eligibility criteria were applied by two reviewers (VAW, MDH) independently to titles and abstracts of retrieved articles. Each reviewer was blinded to the screening of the other reviewer. Papers that met inclusion criteria at the title and abstract screening stage entered a further stage of selection. Full text of these articles was obtained, and studies were screened applying the same eligibility criteria.

Disagreements were resolved by discussion with a third reviewer (BII) and the rest of the review team.

### Data extraction and analysis

Two reviewers (VAW, MDH) extracted data independently into ad hoc developed data extraction forms using Microsoft Excel. The following data were abstracted from all included studies:

- Study design
- Tumor site/location
- Number of reported cases of each tumor type included in the study
- Number of reported cases of each tumor type studied molecularly
- Method(s) of molecular analysis used
- Number of cases with rearrangement of the *MAML2* gene
- Number of cases with *EGFR* mutation, amplification or polysomy
- Number of cases with *KRAS* mutation
- Number of cases with *ERBB2* mutation or amplification
- Types of mutations identified

Two reviewers (VAW, MDH) assessed the risk of bias using an adaptation of the critical appraisal tool for case reports/series developed by the Joanna Briggs Institute (JBI)^26,27^. The JBI critical appraisal tool acknowledges the high risk of bias inherent to the case series study design but allows a general assessment of the methodological quality of a case report or series that captures small methodological differences within this study design and allows for determination of the extent to which a study has addressed the possibility of bias in its design, conduct and analysis. Such an evaluation allowed us to better assess the large number of retrieved reports and explore potentially relevant differences in the quality of this type of research. Our ad hoc adapted version of the tool added an additional criterium to evaluate potential sources of bias relevant to studies of molecular pathology and allows an overall assessment based on a scale of 1–10 (Table 1 and S-Table 2).

**Table 1:** Adapted Joanna Briggs Institute Critical Appraisal Checklist for Case Series.

Were the types of tumors included accurately described?
Were the pathologic features used to diagnose the tumors adequately described?
Did the case series have consecutive inclusion of participants?
Did the case series have complete inclusion of participants?
Was there clear reporting of the demographics of the participants in the study? Age, sex of each patient in a table.
Was there clear reporting of clinical information of the participants? At least body site, TNM, previous treatment reported.
Were the outcomes or follow-up results of cases clearly reported? NA for this study
Were the molecular tests performed adequately described?
Were the mutations and/or other molecular results described in detail?
Were simple descriptive statistics, proportion, differences between groups provided?
Source: *Adapted from https://joannabriggs.org/sites/default/files/2020-08/Checklist_for_Case_Series.pdf

Results are provided as the number and proportion of a tumor type reported to have a specific molecular abnormality in relation to the site of occurrence. Whenever possible a comparison between frequency of a mutation in MEC and ASC is provided.

## Results

**Fig. 1:**
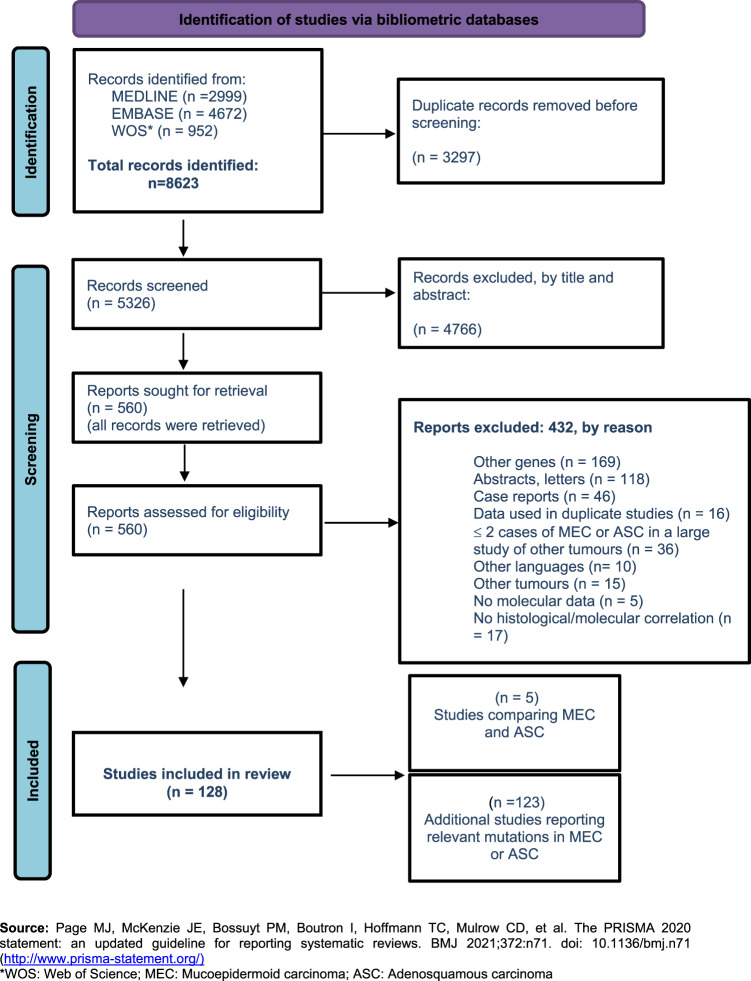
PRISMA 2020 flow diagram for systematic review “Mucoepidermoid carcinoma and adenosquamous carcinoma: the same or different? A systematic review of molecular pathology to aid in classification.”. WOS Web of Science, MEC Mucoepidermoid carcinoma, ASC Adenosquamous carcinoma.

We retrieved a total of 8623 articles, which were reduced to 5326 after duplicates were removed. Applying inclusion and exclusion criteria, we excluded 4766 articles based on title and abstract, and a further 432 based on assessment of the full manuscript, obtaining a final set of 128 included studies that matched our eligibility criteria (Fig. 1).

Five publications^22,28–31^ investigated both tumors in the same study precisely answering our review question (Table 2). These studies used comparable methods to examine both tumors and the reported outcomes provide descriptive evidence for our review question. The remaining 123 studies (references provided in S-Table 4 of Supplementary Materials) reported on *MAML2* rearrangement and mutations of three other commonly affected genes for either of the tumor types (*EGFR, KRAS, ERBB2*).

**Table 2:** Summary of findings of included studies (all case series) analyzing *MAML2* rearrangement in adenosquamous carcinoma and mucoepidermoid carcinoma in the same study.

Author, year	Location	Body site, sample size	Population (gender; age in years)	Relevant molecular determinations				Methodological quality^c^
				ASC	ASC	MEC	MEC	
				*CRTC1-MAML2* fusion by RT-PCR (+/total)	*MAML2* RA by FISH (+/total)	*CRTC1-MAML2* fusion by RT-PCR (+/total)	*MAML2* RA by FISH (+/total)	
Saeki et al.^31^	Japan	Pancreas, *n* = 37 ASC^a^	M**:**20F**:**17 Age: 12 patients <65; 25 patients ≥ 65	0/37	0/12	NA	NA	High
		Salivary gland, *n* = 20 MEC	M**:**8F**:**12 Age: 18 patients < 65; 2 patients ≥ 65	NA	NA	11/20	ND	
Achcar et al.^28^	USA	Lung, *n* = 98 (16 ASC, 17 MEC)	MEC patients M:5F**:**12 Age: 23–82 (mean 45) No data ASC	0/16	0/16	6/14	13/17	Moderate
Zhu et al.^22^	China	Lung, *n* = 82 (40 ASC, 42 MEC)	MEC patients M:25F**:**17 Age: 14–76 (median 53.5) No data ASC	ND	0/40	ND	21/42	Moderate
Lennerz et al.^29^	USA	Uterine cervix, *n* = 21 (14 ASC, 7 MEC)	MEC patients F: 7 Age: 28–88 (median 59) No data ASC	0/14	0/14	1/7	5/7^b^	Moderate
Roden et al.^30^	USA	Thymus, *n* = 5 (3 ASC, 2 MEC)	MEC patients M: 1F**:** 1 Age: 38, 68 No data ASC	ND	0/3	ND	2/2	Moderate

*ASC* adenosquamous carcinoma, *MEC* mucoepidermoid carcinoma, *CRTC1* CREB regulated transcription coactivator 1 gene, *MAML2* mastermind-like transcriptional coactivator 2, *(+/total)* positive cases of total reported cases, *M* male, *F* female, *FISH* Fluorescence in Situ Hybridization, *NA* not applicable, *ND* not done, *No data ASC* No demographics/population data for ASC cases reported, *RA* Rearrangement, *RT-PCR* reverse-transcription-polymerase chain reaction.

^a^Including 16 pancreatic ASC with MEC-like features.

^b^Including 3 cases showing CRTC1 RA, but not MAML2 RA.

^c^Methodological quality of included case series assessed using an adapted version of the JBI Critical Appraisal Tool for case series (^34,35^).

The five studies directly comparing MEC and ASC were all case series, reporting on a range of 5–82 cases of ASC and MEC combined (Table 2).

Three studies were performed in the USA^28–30^, one in Japan^31^ and one in China^22^. All reported only aggregated demographic data, making it difficult to describe the patients fully. Two studies used both RT-PCR and FISH to determine *MAML2* rearrangement in ASC and MEC^28,29^, two studies used FISH only and one study used both techniques to assess ASC^22,30^, but only FISH to assess MEC^31^. Only one^31^ of the five studies was evaluated as having a low risk of bias (Table 2 and supplementary material S-Table 3): a retrospective case series describing 37 pancreatic ASC that included 12 tumors with MEC-like features with the aim of clarifying whether pancreatic ASC with salivary gland-type MEC-like morphology is a pancreatic counterpart of salivary gland MEC. The other four studies were considered to have a moderate risk of bias, the main reasons being a lack of inclusion of all and/or consecutive tumors and lack of presentation of individual, disaggregated patient data. The methods for diagnosis of tumors and the reporting of molecular features were adequately described in all five studies.

**Fig. 2:**
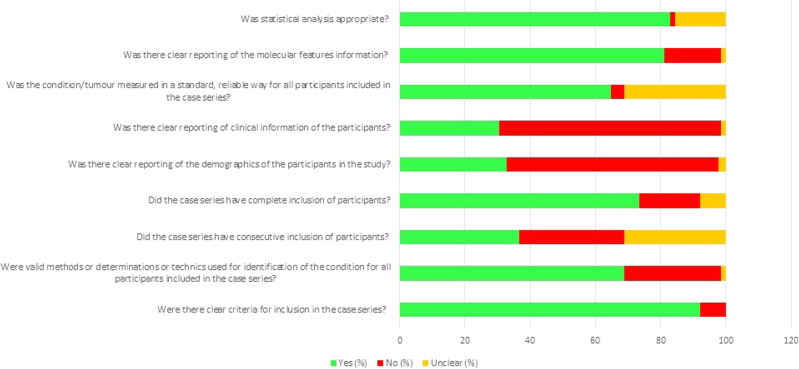
Adapted criteria of the Joanna Briggs Institute Critical Appraisal Tool for 128 included studies (all case series) in percentages.

Of the 123 remaining articles reporting on one tumor type only, all except one, were case series (Table 3 and S-Table 4). One study had a case control design^32^ comparing ASC with conventional squamous cell carcinoma of the head and neck region. Again, most studies reported incomplete and/or aggregated data for variables such as demographics and staging, making comparison by individual cases impossible (Fig. 2 and S-Table 3). The sample size of the original studies from which the tumors of interest were taken ranged from 2 to 21,445, but often the tumors of interest were only a small subset of a larger pool of tumors investigated, most frequently non-small cell carcinomas of lung. The number of cases of MEC or ASC in the individual studies ranged from 2 to 631, and the number studied by molecular techniques was often less.

**Table 3:** Summary of findings for all studies.

Groups of studies examing *MAML2* rearrangement^a^	Number of studies	Number of cases included (range)	Sites examined	Number of reported mutations			
				MEC (*n*)	%	ASC (*n*)	%
1. *MAML2*	5	5–82	Pancreas, salivary glands, lung, cervix, thymus	52/88	59.1	0/110	0
2. *MAML2*	49	2–217	Salivary gland, lung, thyroid, skin, head & neck, breast	1160/1715	67.6	0/37	0
3. *MAML2*	4	9–101	Salivary gland, lung	109/172	63.4		
TOTAL MAML2 rearrangment				1337/2009	66.6	0/147	0
Other genes							
*EGFR*	43	2–631	Salivary gland, lung, pancreas, cervix, esophagus, gall bladder	11/329	3.3	660/1795	36.8
*KRAS*	31	8–101	Salivary gland, lung, pancreas, cervix, gall bladder	11/266	4.1	143/625	22.9
*ERBB2*	10	3–76	Salivary gland, lung, pancreas	9/126	7.1	6/196	3.1

^a^As grouped in Results section of text

The 123 included studies reporting on one tumor type (S-Table 4) used the following methods of molecular investigation: 1) Rearrangement of the *MAML2* gene identified by FISH, or targeted RT-PCR followed by sequencing. A few older studies used conventional cytogenetics. 2) *EGFR*, *KRAS*, and *ERBB2* mutations identified by targeted RT-PCR of specific exons followed by sequencing, by MALTI-TOF mass spectroscopy or by next-generation sequencing techniques. 3) *EGFR* amplification/polysomy or *ERBB2* amplification identified by FISH, comparative genomic hybridization, or next-generation sequencing.

### Mutations

**Fig. 3:**
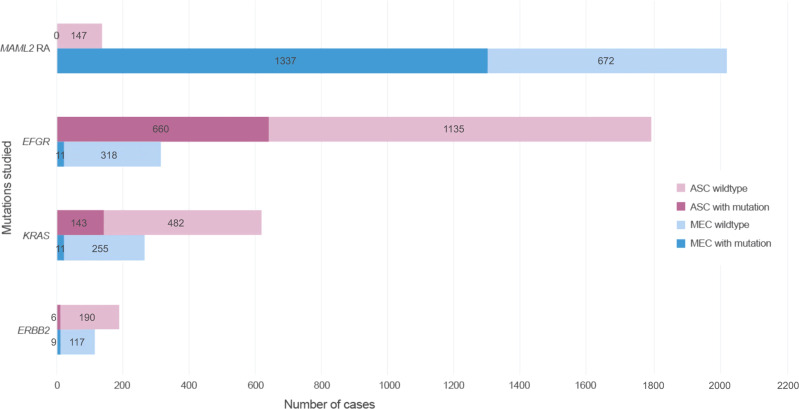
Number of reported mutations and wildtype cases by tumor type in the 128 included studies.

*MAML2* gene rearrangement was the most frequent molecular alteration encountered in MEC, seen in 1337/2009 (66.6%) of cases (Table 3 and Fig. 3), followed by mutations in *ERBB2* (9/126, 7.1%), *KRAS* (11/266, 4.1%), and *EGFR* (11/329 cases, 3.3%). No *MAML2* gene rearrangements were found in 147 ASC. Instead, ASC tumors harbored mutations of *EGFR* in (660/1705, 38.7%) of cases, *KRAS* in (143/625, 22.9%), and *ERBB2* in (6/196, 3.1%). *KRAS* mutations were reported in (108/126, 85.7%) pancreatic ASC.

The studies reporting the above mutations were separated into four categories: 1) studies examining *MAML2* rearrangement in both MEC and ASC, 2) studies searching for *MAML2* rearrangement in MEC only or ASC only, 3) studies examining *EGFR*, *KRAS* and/or *ERRB2* molecular abnormalities in MEC only; and 4) studies examining *EGFR*, *KRAS* and/or *ERRB2* molecular abnormalities in ASC only (Supplementary materials, S-Table 4).

#### *MAML2* rearrangement determined in MEC and ASC in the same study

The five included studies that investigated *MAML2* rearrangement in both -MEC and ASC in the same tumor set examined a total of 88 MEC and 110 ASC in pancreas, salivary glands, lung, cervix and thymus (Tables 2 and 3). Not all tumors were tested by both methods, without stated reasons.

Overall, 52 of 88 MEC cases (59%) had *MAML2* rearrangement detected (18/55 by RT-PCR and 41/68 by FISH), whereas none of 110 ASC studied showed this rearrangement. Five studies used *MAML2* break-apart probes, while 1 study also used a *CRTC1* break-apart probe^29^.

#### *MAML2* rearrangement reported in MEC only or ASC only

45 of 123 (36.6%) of the studies included in the review examined *MAML2* rearrangement in MEC only, and 4 studies examined *MAML2* rearrangement in ASC of the head and neck and lung (Table 3 and Supplementary materials S-Table 4). Of those, 34 studies comprising 1569 cases examined exclusively salivary gland MECs while 11 studies comprising 148 cases studied MEC from multiple sites, including salivary gland, lung, skin, breast, oropharynx, thyroid, and jaw. The presence of *MAML2* rearrangement was confirmed by 1) traditional cytogenetics in 16/34 cases (47%); 2) by RT-PCR in 400/611 (65.5%) salivary gland MECs, and in 70/99 (70%) MECs from mixed sites; and 3) by FISH in 763/1150 (66.3%) of salivary gland MECs and in 36/37 (97.3%) from the mixed group. In addition, *MAML2* rearrangement was also documented in MEC in four additional studies, which primarily looked for other mutations (group 3) resulting in another 109/172 (63.4%) cases with *MAML2* rearrangement, (101/152 positive by FISH and 97/154 by RT-PCR or NGS). Thus, the total number of MECs with *MAML2* rearrangement was 1337/2009 (66.6%). The studies that looked for *MAML2* rearrangement in 37 cases of ASC found none^32^.

#### *EGFR*, *KRAS,* and/or *ERRB2* molecular abnormalities reported in MEC only

*EGFR*, *KRAS,* and/or *ERRB2* abnormalities were examined in MEC in 19 of the 123 studies (15.4%) (Table 3 and Supplementary materials S-Table 4). This group comprised 335 patients in 14 studies of salivary gland tumors and 101 patients in 5 studies of lung tumors. *EGFR* gene mutations were identified in 4/262 (1.5%) salivary gland MECs and 7/67 (10.4%) lung MECs (total 11/329, (3.3%). Cytogenetic studies showed *EGFR* polysomy in 11/23 salivary gland MECs and 2/12 lung MECs, for a total of 13 of 35 tumors (37.1%). *EGFR* gene amplification was seen in 9/127 (7.1%) salivary gland MECs and 0/12 (0%) lung MECs. *KRAS* gene mutations were found in 11/257 (4.3%) salivary gland MECs and 0/9 (0%) lung MECs, for a total of 11/266 (4.1%). *ERBB2* gene amplification or mutation was documented in 9/126 (7.1%) salivary gland MECs. No lung MECs were studied for *ERBB2* amplification.

#### *EGFR*, *KRAS*, and/or *ERRB2* molecular abnormalities reported in ASC

Among the 123 selected studies, 55 (44.7%) examined *EGFR*, *KRAS,* and/or *ERRB2* molecular abnormalities in ASC (Table 3 and Supplementary materials, S-Table 4). Of these, 36 examined *EGFR* mutation in lung tumors, 4 in pancreas and 1 each in cervix, esophagus, and gallbladder. *EGFR* mutations were detected in 660/1705 (38.7%) lung ASC, whereas as none of 30 cervix, 58 pancreas, or 2 gallbladder ASC showed these mutations. There were 17 studies focusing on *KRAS* mutations in lung tumors, 10 in pancreas, 3 in cervix, and 1 in gallbladder. *KRAS* mutations were identified in 108/126 (85.7%) pancreatic ASC, 30/338 (8.9%) lung ASC, 5/159 (3.1%) cervical ASC, and none of the two gallbladder ASCs. In addition, 8 studies of lung ASCs and 2 of pancreas searched for *ERBB2* mutations. *ERBB2* mutations were demonstrated in 6/147 (4.1%) lung tumors, but none of 31 pancreas, 16 esophagus, or 2 gallbladder ASCs. Cervical ASCs were not investigated for *ERBB2* mutations. All mutations (where provided in the publication) are listed in Supplementary materials, (S-Table 5).

## Discussion

We applied systematic review methodology to the field of pathology to summarize available evidence from studies on molecular alterations in MEC and ASC. We focused on those two tumors as they have overlapping histopathological features leading to the possibility of misdiagnosis^33^. The advantage of focusing on these two tumor types is that MEC has a single molecular abnormality, *t*(11;19), resulting in rearrangement of the *MAML2* gene, which is thought to be specific for MEC^34^, unlike many other gene fusions and mutations. We found that 66.6% of retrieved cases histologically diagnosed as MEC showed this abnormality, and that it was not present in any ASCs, supporting the classification of these two tumors as distinct entities. Moreover, *MAML2* rearrangement is seen consistently in MECs from various anatomical sites and in a broadly similar proportion of MEC cases, suggesting that MECs of various sites may be considered as one entity.

In contrast to MEC, ASCs from different anatomical sites show distinct differences in their molecular profiles with only lung ASCs showing *EGFR* mutations and pancreatic ASCs almost invariably showing *KRAS* mutations^35–37^.

We found that ASC of lung has similar genetic abnormalities as those of adenocarcinoma of the lung, with about a third of the cases harboring an *EGFR* mutation. In contrast fewer than 5% of MECs have *EGFR* mutation. Similarly, the finding that 85.7% of pancreatic ASC had mutations in the *KRAS* gene confirms its resemblance to pancreatic adenocarcinoma, where nearly 100% have *KRAS* mutations^38,39^. Only a small proportion of ASC in other sites show this mutation^40,41^, which means that it cannot be considered a distinctive molecular feature of this tumor in general, nor used for classification purposes outside the pancreas.

One limitation of our review is the scant number of included studies comparing MEC and ASC head-to-head, which forced us to rely on studies analyzing only one of these tumor types at a time, with the resulting heterogeneity of study designs, molecular techniques and case selection leading to a moderate/high risk of bias. The five studies that did include both MEC and ASC in the same study did not investigate other molecular alterations, therefore we could not find sufficient evidence to describe a distinct molecular signature of *MAML2*-negative MEC as compared to ASC. Clearly, more research is needed to address this specific aim.

What molecular abnormalities do the one third of MECs without *MAML2* rearrangement have and are they really MEC? Lennerz et al. found in his case series that three of five cases of MEC of cervix had rearrangement of the *CRTC1* gene, but not *MAML2*, and 3 cases had amplification of *MAML2*, suggesting that other mechanisms may lead to tumorigenesis in those MECs without the canonical *CRTC1/3*::*MAML2* fusions^29^. Similarly, the same group, found *CRTC1* rearrangement in MEC of the skin by FISH, but did not find *CRTC1*::*MAML2* fusion by RT-PCR^42^. No other studies of MEC used FISH of the *CRTC1* gene, so it is unknown what proportion of MECs that do not show *MAML2* rearrangement might have *CRTC1* rearrangement that involves another gene partner. Kang et al. found mutations of p53 in five of nine high-grade MECs^43^, but no other recurrent abnormality has been identified. This raises the possibility that tumors classified as high-grade MECs are actually ASC or another tumor, yet to be defined; however, low grade MECs may also be negative for *MAML2* rearrangement. The impressive variety of genes investigated suggests there is a significant biological heterogeneity of molecular alterations encountered in the two tumor types, which has yet to be fully explored. The included studies did not allow examination of alterations present in the *MAML2* rearrangement-negative subgroup of MECs.

When considering implications of the results of our systematic review to routine pathology practice, they point to utility in determining the presence of *MAML2* rearrangement to confirm the diagnosis of MEC. A negative result for *MAML2* rearrangement is less informative, as both tumor types can be negative, which limits the potential diagnostic value of this test. Tumors without *MAML2* rearrangement still need to be classified as MEC or ASC based on morphology. Although molecular alterations in *EGFR* and *KRAS* occur more frequently in ASC than in MEC, we did not find evidence of these changes being either specific or sensitive enough to use diagnostically and are therefore of little value in tumor classification at this moment. Their value may instead be in predicting response to tyrosine kinase inhibitors, though this would require clinical trial data.

All our conclusions rely on information from case series, which ideally should only be considered as hypothesis-generating research. Therefore, there is a need for higher level studies that apply an appropriate study design to confirm these findings and to characterize the relationship (Table 4). Such research will need to address methodological requirements, such as sample selection, statistical analyses, risk of bias, etc. to assure the internal and external validity of the study and to prove an association between a distinctive molecular profile and MEC or ASC. One methodologically suitable option would be a diagnostic cohort study that investigates the determination of the mutation in a prospective blind comparison with the diagnostic reference standard (histopathology) in a consecutive series of patients from a representative clinical population^44,45^. However, the preferred design and gold standard should be a diagnostic randomized controlled trial, a design that is rarely applied in diagnostic research, but would produce clinically relevant outcomes and permit better informed decisions on issues relevant to classification and diagnosis. Such trials are randomized comparisons of two diagnostic investigations (one standard and one experimental) with identical therapeutic intervention, analyzing outcomes that are clinically important consequences of diagnostic accuracy^45,46^. While the diagnostic cohort study could provide evidence on the relative accuracy of such a mutation, the clinical trial would add information on the clinical relevance of diagnostic accuracy and its impact on patient outcomes. In Table 4 we provide a short summary of the characteristics of studies required to improve the current knowledge on the molecular profile of MEC and ASC, which would help to avoid the most commonly detected biases.

**Table 4:** Methodological considerations relevant to determination of the molecular profile of tumors.

A. Application of an adequate study design			
Study design	Outcome measures	Pros	Cons
Diagnostic randomized controlled trials: adequately controlled with hypotheses stated in advance and evaluated according to a standardized protocol	Sensitivity, specificity, likelihood ratios, accuracy	Properly conducted, randomized controlled trials are the gold standard to determine accuracy, safety and effectiveness of diagnostic tests. Permit analysis by “intention-to-test” and control of biases, such as context and clinical review bias	Needs more resources, sample size is relevant, and interdisciplinary teamwork required.
Diagnostic cohort studies: allow assessment of the characteristics of a diagnostic test, with control group	Sensitivity and specificity, PPV and NPV, likelihood ratios, diagnostic odd ratios and accuracy can be calculated	Relatively inexpensive, simple to perform, well accepted among the medical research community.	Not directly tied to patient outcomes, risk of bias inherent to study design and difficult to control for confounders and interactions.
Prospective case series: large, consecutive selected, prospective case series		Inexpensive, simple to perform, well accepted among clinical community.	Allows only weak inferences and high likelihood of bias associated.
B. Potential confounders to take into account during the statistical analysis			
Related to	To be taken into account for adjustment		
Determination of the molecular alteration: laboratory methods and technics	Heterogeneity in techniques, different cut offs, variation in measurements, not comparable values provided, geographical differences in determination methods, fast advancing technology with differences over time in determinations		
Study population: method of ample selection and sample size	Potential study participants with confounders known to influence experimental test accuracy excluded from study		
Test performance: indeterminate tests/Not performed test	Performance of a diagnostic test may vary in different settings (each setting, different mix of patients)		
C. Main biases to avoid			
Type of bias	Step in research in which bias control can be applied		
Selection bias	Study design		
Reporting bias	Analysis and dissemination		
Context bias:	Study design		
Clinical review bias:	Study design		

The potential biases detected in studies included in this review are:

1. Sample size and selection: Small sample sizes or low numbers of reported MEC or ASC in the tumors included in a study was a common problem and reduced the representativeness of the reported results, already compromised by the case series design and retrospective selection of participants. In many of the studies, particularly those involving lung carcinomas, ASC were only a small percentage of the total number of tumors investigated and were not the main focus of investigation. This makes it impossible to use their results to derive definitive conclusions.
2. Data collection: All of the studies were of a retrospective nature.
3. Study design: All, except one study, were case series, a study type with an inherent high risk of bias. Only one study concerning ASC and squamous carcinoma of the head and neck region used a case control design, trying to provide association measures and to control for potential confounders.
4. Analysis (molecular and statistical): Most studies didn’t perform a complete analysis of all specimens, increasing the reporting bias. Only a fraction of the cases of each tumor type provided actual molecular results, causing analysis, interpretation and reproducibility of the results to be compromised.
5. Reporting: In most studies demographic, clinical and tumor variables, such as age, sex, TNM stage, treatment or outcome by molecular results were not disaggregated by individual cases, compromising comparability and reproducibility of results.

In conclusion, by standards of systematic review methodology and despite the large number of retrieved studies, we did not find adequate evidence for a distinctive molecular profile of either MEC or ASC. Reported cases retrieved in this review point towards the relevance of *MAML2* rearrangement in confirming the diagnosis of MEC when positive, but this finding needs to be proven by well-designed studies with accurate statistical analysis. Other aspects that need to be studied include *MAML2* rearrangement negative MEC cases and their underlying molecular alterations.

Regarding ASC, we did not find any cases that had *MAML2* rearrangement but did not otherwise find a distinctive molecular profile. This review did not find studies that performed detailed molecular analyses of both tumors in the same investigation which would have answered the specific review question, instead retrieving only low evidence level studies with a high risk of bias^47,48^.

We have shown that systematic review methods can be applied to the field of pathology by summarizing and evaluating the available evidence, at the same time as showing research gaps. Systematic review should be performed more frequently for pathology relevant topics, and we believe that our study is a paradigm for this approach. There is clearly an urgent need for more research and well conducted studies to investigate the molecular profiles of MEC and ASC. Research is needed that considers potential confounders and biases affecting previous work and which applies appropriate methods to control for them in future studies.

## Supplementary information

Supplementary to: Mucoepidermoid carcinoma (MEC) and adenosquamous carcinoma (ASC): the same or different? A systematic review of molecular pathology to aid in classification

Adapted JBI Critical Appraisal Checklist for Case Series for included studies.

Summary of findings table for 123 included studies reporting only on one tumor.

Specific mutations in EGFR, KRAS and ERBB2 identified in MEC and ASC as reported in included references.

## Data Availability

All data generated or analyzed during this study are included in this published article and its supplementary information files.

## References

[1] World Health Organization Classification of Tumours Editorial Board, *BlueBooksOnline - WHO Classification of Tumours Online*, https://tumourclassification.iarc.who.int/aboutus (2021).

[2] Cree, I. A., Indave Ruiz, B. I., Zavidil J., McKay J., Olivier M., Kozlakidis Z., et al. The International Collaboration for Cancer Classification and Research. *Int. J. Cancer* **148**, 560-571 (2021). 10.1002/ijc.33260 PMC7756795 32818326

[3] Hoadley, K. A., Yau, C., Wolf, D. M., Cherniack, A. D., Tamborero D., Ng, S., *et al*. Multiplatform analysis of 12 cancer types reveals molecular classification within and across tissues of origin. *Cell* **158**, 929944 (2014). 10.1016/j.cell.2014.06.049 PMC4152462 25109877

[4] Uttley, L., Indave, B.I., Hyde, C., White, V., Lokuhetty, D. & Cree, I. Invited commentary-WHO Classification of Tumours: How should tumors be classified? Expert consensus, systematic reviews or both? *Int. J. Cancer* **146**, 3516-3521 (2020). 10.1002/ijc.32975 PMC7818407 32170735

[5] Diaz-Cano, S. J. Tumor heterogeneity: mechanisms and bases for a reliable application of molecular marker design. *Int. J. Mol. Sci*. **13**, 1951-2011 (2012). 10.3390/ijms13021951 PMC3292002 22408433

[6] Colomer, R., Mondejar, R., Romero-Laorden, N., Alfranca, A., Sanchez-Madrid, F. & Quintela-Fandino, M. When should we order a next generation sequencing test in a patient with cancer? *eClinicalMedicine* **25**, 100487 (2020). 10.1016/j.eclinm.2020.100487 PMC7397394 32775973

[7] Nemtsova, M. V., Kalinkin, A. I., Kuznetsova, E. B., Bure, I. V., Alekseeva, E. A., Khorobrykh, T. V., *et al*. Clinical relevance of somatic mutations in main driver genes detected in gastric cancer patients by next-generation DNA sequencing. *Sci. Rep*. **10**, 504 (2020). 10.1038/s41598-020-57544-3 PMC6965114 31949278

[8] Altman, N. & Krzywinski, M. Association, correlation and causation. *Nat. Methods* **12**, 899-900 (2015). 10.1038/nmeth.3587 26688882

[9] Schober, P., Boer, C. & Schwarte, L. A. Correlation Coefficients: Appropriate Use and Interpretation. *Anesth. Analg.* **126**, 1763-1768 (2018). 10.1213/ANE.0000000000002864 29481436

[10] Biomarkers and surrogate endpoints: preferred definitions and conceptual framework. *Clin. Pharmacol. Ther.* **69**, 89-95 (2001). 10.1067/mcp.2001.113989 11240971

[11] Strimbu, K. & Tavel, J. A. What are biomarkers? *Curr. Opin. HIV AIDS* **5**, 463466 (2010). 10.1097/COH.0b013e32833ed177 PMC3078627 20978388

[12] World Health Organization Classification of Tumours Editorial Board, *Thoracic tumours*. 5th edn, Vol. 1 (IARC Publications: Lyon (France), 2019).

[13] Keelawat, S., Liu, C. Z., Roehm, P. C. & Barnes, L. Adenosquamous carcinoma of the upper aerodigestive tract: a clinicopathologic study of 12 cases and review of the literature. *Am. J. Otolaryngol*. **23**, 160-168 (2002). 10.1053/ajot.2002.123462 12019485

[14] Jordan, E. J., Basturk, O., Leach, S.D., Klimstra, D.S., Allen, P.J., Berger, M.F., *et al*. Clinical and Molecular Analysis of Adenosquamous Carcinoma of the Pancreas. *J. Pancreas* **17**, 620-628 (2016).

[15] Alos, L., Castillo, M., Nadal, A., Caballero, M., Mallofre, C., Palacin, A., *et al*. Adenosquamous carcinoma of the head and neck: criteria for diagnosis in a study of 12 cases. *Histopathology* **44**, 570-579 (2004). 10.1111/j.1365-2559.2004.01881.x 15186272

[16] El-Naggar A.K., Chan, J. K. C., Grandis, J.R., Takata, T. & Slootweg, P.J. *WHO Classification of Head and Neck Tumours*. 4th edn, Vol. 1 (IARC Publications, 2019).

[17] Nordkvist, A., Gustafsson, H., Jubergode, M. & Stenman, G. Recurrent rearrangements of 11Q14-22 in Mucoepidermoid Carcinoma. *Cancer Genet*. **74**, 77-83 (1994). 10.1016/0165-4608(94)90001-9 8019965

[18] Enlund, F., Behboudi, A., Andren, Y., Oberg, C., Lendahl, U., Mark, J., *et al*. Altered Notch signaling resulting from expression of a WAMTP1-MAML2 gene fusion in mucoepidermoid carcinomas and benign Warthin’s tumors. *Exp. Cell Res*. **292**, 21-28 (2004). 10.1016/j.yexcr.2003.09.007 14720503

[19] Bell, D. & El-Naggar, A. K. Molecular Heterogeneity in Mucoepidermoid Carcinoma: Conceptual and Practical Implications. *Head and Neck Pathol*. **7**, 23-27 (2013). 10.1007/s12105-013-0432-5 PMC3597160 23459841

[20] Bishop, J. A., Cowan, M. L., Shum, C. H. & Westra, W. H. MAML2 Rearrangements in Variant Forms of Mucoepidermoid Carcinoma: Ancillary Diagnostic Testing for the Ciliated and Warthin-like Variants. *Am. J. Surg. Pathol*. **42**, 130-136 (2018). 10.1097/PAS.0000000000000932 PMC5730480 28877061

[21] Martins, C., Cavaco, B., Tonon, G., Kaye, F. J., Soares, J. & Fonseca, I. A study of MECT1-MAML2 in mucoepidermoid carcinoma and Warthin’s tumor of salivary glands. *J. Mol. Diagn.* **6**, 205-210 (2004). 10.1016/S1525-1578(10)60511-9 PMC1867632 15269296

[22] Zhu, F., Wang, W., Hou, Y., Shi, J., Liu, Z., He D., *et al*. MAML2 Rearrangement in Primary Pulmonary Mucoepidermoid Carcinoma and the Correlation with FLT1 Expression. *Plos One* **9** (2014). 10.1371/journal.pone.0094399 PMC3979848 24714697

[23] Jee, K. J., Persson, M., Heikinheimo, K., Passador-Santos, F., Aro, K., Knuutila, S., *et al*. Genomic profiles and CRTC1-MAML2 fusion distinguish different subtypes of mucoepidermoid carcinoma. *Mod. Pathol.* **26**, 213222 (2013). 10.1038/modpathol.2012.154 23018873

[24] Moher, D., Shamseer, L., Clarke, M., Ghersi, D., Liberati,A., Petticrew, M., *et al*. Preferred reporting items for systematic review and metaanalysis protocols (PRISMA-P) 2015 statement. *Syst. Rev.* **4**, 1-1 (2015). 10.1186/2046-4053-4-1 PMC4320440 25554246

[25] Page, M. J., McKenzie, JE., Bossuyt, P. M., Boutron, I., Hoffman, T. C., Mulrow, C. D., *et al*. The PRISMA 2020 statement: an updated guideline for reporting systematic reviews. *BMJ* **372**, n71 (2021). 10.1136/bmj.n71 PMC8005924 33782057

[26] Moola, S., Munn, Z., Sears, K., Sfetcu, R., Currie, M., Lisy, K. *et al*. Conducting systematic reviews of association (etiology): The Joanna Briggs Institute’s approach. *Int. J. Evid. Based Healthc.* **13**, 163169 (2015). 10.1097/XEB.0000000000000064 26262566

[27] Moola, S., Munn, Z. T. C., Aromataris, E., Sears, K., Sfetcu, R., Currie, M., *et al*. Chapter 7: Systematic reviews of etiology and risk. In: Aromataris E and Munn Z (ed). *Joanna Briggs Institute Reviewer’s Manual*. (The Joanna Briggs Institute, 2017).

[28] Achcar Rde, O., Nikiforova, M. N., Dacic, S., Nicholson, A. G. & Yousem, S. A. Mammalian mastermind like 2 11q21 gene rearrangement in bronchopulmonary mucoepidermoid carcinoma. *Hum. Pathol.* **40**, 854-860 (2009). 10.1016/j.humpath.2008.11.007 19269006

[29] Lennerz, J. K., Perry, A., Mills, J. C., Huettner, P. C. & Pfeifer, J. D. Mucoepidermoid carcinoma of the cervix: another tumor with the t(11;19)-associated CRTC1-MAML2 gene fusion. *Am. J. Surg. Pathol.* **33**, 835843 (2009). 10.1097/PAS.0b013e318190cf5b 19092631

[30] Roden, A. C., Erickson-Johnson, M. R., Yi, E. S. & Garcia, J. J. Analysis of MAML2 rearrangement in mucoepidermoid carcinoma of the thymus. *Hum. Pathol.* **44**, 2799-2805 (2013). 10.1016/j.humpath.2013.07.031 24134933

[31] Saeki, K., Ohisho,Y., Matsuda, R., Mochidome, N., Miyasaka, Y., Yamamoto, H., *et al*. “Pancreatic Mucoepidermoid Carcinoma” Is not a Pancreatic Counterpart of CRTC1/3-MAML2 Fusion Gene-related Mucoepidermoid Carcinoma of the Salivary Gland, and May More Appropriately be Termed Pancreatic Adenosquamous Carcinoma With Mucoepidermoid Carcinoma-like Features. *Am. J. Surg. Pathol*. **42**, 14191428 (2018). 10.1097/PAS.0000000000001135 30138216

[32] Kass, J. I., Lee, S. C., Abberbock, S., Seethala, R. R. & Duvvuri, U. Adenosquamous carcinoma of the head and neck: Molecular analysis using CRTC-MAML FISH and survival comparison with paired conventional squamous cell carcinoma. *Laryngoscope* **125**, E371-E376 (2015). 10.1002/lary.25519 26255977

[33] Chenevert, J., Barnes, L. E. & Chiosea, S. I. Mucoepidermoid carcinoma: A five-decade journey. *Virchows Arch*. **458**, 133-140 (2011). 10.1007/s00428-011-1040-y 21243374

[34] O’Neill, I. D. t(11;19) translocation and CRTC1-MAML2 fusion oncogene in mucoepidermoid carcinoma. *Oral Oncol*. **45**, 2-9 (2009). 10.1016/j.oraloncology.2008.03.012 18486532

[35] Colombino, M., Paliogiannis, P., Cossu, A., Santeufemia, D. A., Sardinian Lung Cancer (CLC) Study Group, Sini, M. C., *et al*. EGFR, KRAS, BRAF, ALK, and cMET genetic alterations in 1440 Sardinian patients with lung adenocarcinoma. *BMC Pulm. Med.* **19**, 209 (2019). 10.1186/s12890-019-0964-x PMC6849322 31711449

[36] Kim, J., Jang, S. J., Choi, C. M. & Ro, J. Y. Correlation of Histologic Subtypes and Molecular Alterations in Pulmonary Adenocarcinoma: Therapeutic and Prognostic Implications. *Adv. Anat. Pathol.* **23**, 330-338 (2016). 10.1097/PAP.0000000000000121 27403614

[37] Rodriguez, E. F., Jones, R., Morris, C. P., Ettinger, D., Chowsilpa, S. & Maleki, Z. *et al*. Molecular Alterations in Pulmonary Adenocarcinoma of African Americans. *Am. J. Clin. Pathol*. **152**, 237-242 (2019). 10.1093/ajcp/aqz038 31114847

[38] Löhr, M., Klöppel, G., Maisonneuve, P., Lowenfels, A. B. & Lüttges, J. Frequency of K-ras mutations in pancreatic intraductal neoplasias associated with pancreatic ductal adenocarcinoma and chronic pancreatitis: a meta-analysis. *Neoplasia (New York, N.Y.)* **7**, 17-23 (2005). 10.1593/neo.04445 PMC1490318 15720814

[39] Timar, J. & Kashofer, K. Molecular epidemiology and diagnostics of KRAS mutations in human cancer. *Cancer Metastasis Rev*. **39**, 1029-1038 (2020). 10.1007/s10555-020-09915-5 PMC7680318 32725342

[40] Cros, J., Sbidian, E., Hans, S., Roussel, H., Scotte, F., Tartour, E., *et al*. Expression and mutational status of treatment-relevant targets and key oncogenes in 123 malignant salivary gland tumours. *Ann. Oncol*. **24**, 2624-2629 (2013). 10.1093/annonc/mdt338 23933559

[41] Kardon, D. E., Thompson, L. D. R., Przygodzki, R. M. & Heffess, C. S. Adenosquamous Carcinoma Of The Pancreas: A Clinicopathologic Series Of 25 Cases. *Mod. Pathol.* **14**, 443-451 (2001). 10.1038/modpathol.3880332 11353055

[42] Lennerz, J. K., Perry, A., Dehner, L. P., Pfeifer, J. D. & Lind, A. C. CRTC1 rearrangements in the absence of t(11;19) in primary cutaneous mucoepidermoid carcinoma. *Br. J. Dermatol*. **161**, 925-929 (2009). 10.1111/j.1365-2133.2009.09200.x 19438452

[43] Kang, H., Tan, M., Bishop, J. A., Jones, S., Sausen, M., Ha, P.K., *et al*. Whole-exome sequencing of salivary gland mucoepidermoid carcinoma. *Clin. Cancer Res*. **23**, 283-288 (2017). 10.1158/1078-0432.CCR-16-0720 PMC5182193 27340278

[44] Jaeschke, R., Guyatt, G. H. & Sackett, D. L. Users’ guides to the medical literature. III. How to use an article about a diagnostic test. B. What are the results and will they help me in caring for my patients? The EvidenceBased Medicine Working Group. *JAMA* **271**, 703-707 (1994). 10.1001/jama.271.9.703 8309035

[45] Rodger, M., Ramsay, T. & Fergusson, D. Diagnostic randomized controlled trials: the final frontier. *Trials* **13**, 137-137 (2012). 10.1186/1745-6215-13-137 PMC3495679 22897974

[46] Lijmer, J. G. & Bossuyt, P. M. Various randomized designs can be used to evaluate medical tests. *J. Clin. Epidemiol.* **62**, 364-373 (2009). 10.1016/j.jclinepi.2008.06.017 18945590

[47] Burns, P. B., Rohrich, R. J. & Chung, K. C. The levels of evidence and their role in evidence-based medicine. *Plast. Reconstr. Surg.* **128**, 305-310 (2011). 10.1097/PRS.0b013e318219c171 PMC3124652 21701348

[48] Murad, M. H., Asi, N., Alsawas, M. & Alahdab, F. New evidence pyramid. *J. Evid. Based Med*. **21**, 125 (2016). 10.1136/ebmed-2016-110401 PMC4975798 27339128

