# Supplementary material for: Mucoepidermoid carcinoma (MEC) and adenosquamous carcinoma (ASC), the same or different entities?
Source: Mod Pathol. 2022 Jul 23;35(10):1484–93. doi: 10.1038/s41379-022-01100-z (PMC9514988; doi:10.1038/s41379-022-01100-z)
Supplement: Supplementary file 1 — Supplementary to: Mucoepidermoid carcinoma (MEC) and adenosquamous carcinoma (ASC): the same or different? A systematic review of molecular pathology to aid in classification [file 41379_2022_1100_MOESM1_ESM.pdf]

Supplementary Materials 1

Supplementary to: Mucoepidermoid carcinoma (MEC) and adenosquamous carcinoma (ASC): the same or different? A systematic review of molecular pathology to aid in classification

Authors: Valerie A. White, Martin D. Hyrcza, Jochen K. Lennerz, Julia Thierauf, Dilani Lokuhetty, Ian A. Cree, Blanca Iciar Indave

The supplementary data provides the applied search strategy (S-Table 1-Word document), the criteria applied to score the quality appraisal using an adapted version of the Joanna Briggs Institute Critical Appraisal tool for Case Series (S-Table 2-Word document) and the evaluation of methodological quality (S-Table 3-Excel), the summary of findings table for the 123 included studies that analyzed only one tumour type (S-Table 4-Excel), the table of specific mutations identified in the studies (S-Table 5-Excel) and the PRISMA checklist for this review (S-Table 6-Word).

S-Table 1: Tailored search strategy by bibliometric database

Number	Query	Results
	**MEDLINE**	
1	Carcinoma, Mucoepidermoid[MeSH Terms] OR "MEC"	
2	Mucoepidermoid Tumor[MeSH Terms]	
3	Carcinoma, Adenosquamous[MeSH Terms]	
4	mucoepidermoid	
5	adenosquamous	
6	Molecular Medicine[MeSH Subheading]	
7	Molecular Sequence Data[MeSH Terms]	
8	Genetic Techniques[MeSH Terms]	
9	Epigenomics[MeSH Terms]	
10	DNA Methylation[MeSH Terms]	
11	CRTC1 protein, human[Supplementary Concept]	
12	CRTC3 protein, human[Supplementary Concept]	
13	MAML1 protein, human[Supplementary Concept]	
14	MAML2 protein, human[Supplementary Concept]	
15	MECT1-MAML2 fusion protein[Supplementary Concept]	
16	next generation sequenc*	
17	whole genome sequenc*	
18	whole exome sequenc*	
19	nucleotide sequenc*	
20	"molecular analysis"	
21	"molecular analyses"	
22	"genetic analysis"	
23	"genetic analyses"	
24	DNA sequenc*	
25	chromosome map*	
26	epigenetic test*	
27	"DNA methylation"	

28	"fluorescence in-situ hybridization"	
29	"FISH"	
30	"polymerase chain reaction"	
31	"RT-PCR"	
32	"reverse-transcription PCR" OR	
33	karyotyping	
34	genomic profil*	
35	"mutation analysis"	
36	"mutation analyses"	
37	somatic mutation*	
38	copy number alteration*	
39	"fusion positive"	
40	"fusion negative"	
41	"t(11;19)"	
42	CRTC1	
43	CTRC3	
44	MAML1	
45	MAML2	
46	MECT1	
48	#1 OR #2 OR #3 OR #4 OR #5	
49	#6 OR #7 OR #8 OR #9 OR #10 OR #11 OR #12 OR #13 OR #14 OR #15 OR #16 OR #17 OR #18 OR #19 OR #20 OR #21 OR #22 OR #23 OR #24 OR #25 OR #26 OR #27 OR #28 OR #29 OR #30 OR #31 OR #32 OR #33 OR #34 OR #35 OR #36 OR #37 OR #38 OR #39 OR #40 OR #41 OR #42 OR #43 OR #44 OR #45 OR #46	
50	48 AND 49	**2387**
**EMBASE**		
1	exp mucoepidermoid tumor/	
2	exp adenosquamous carcinoma/	
3	mucoepidermoid.mp.	
4	adenosquamous.mp.	
5	exp molecular genetics/	

6	exp genetic procedures/
7	exp genetic analysis/
8	exp dna sequence/
9	exp epigenetics/
10	exp DNA methylation/
11	exp fluorescence in situ hybridization/
12	exp polymerase chain reaction/
13	exp somatic mutation/
14	exp gene fusion/
15	exp fusion gene/
16	exp chromosome translocation/
17	exp reverse transcription polymerase chain reaction/
18	1((next generation or whole genome or whole exome or nucleotide) adj1 sequenc*).mp. [mp=title, abstract, heading word, drug trade name, original title, device manufacturer, drug manufacturer, device trade name, keyword, floating subheading word, candidate term word]
19	1genetic analys?s.mp.
20	((molecular or genetic) adj1 analys?s).mp.
21	DNA sequenc*.mp.
22	chromosome map*.mp.
23	epigenetic test*.mp.
24	DNA methylation.mp.
25	fluorescence in-situ hybridization.mp.
26	FISH.mp.
27	polymerase chain reaction.mp.
28	RT-PCR.mp.
29	reverse-transcription PCR.mp.
30	karyotyping.mp.
31	31. genomic profil*.mp.
32	mutation analys?s.mp.
33	somatic mutation*.mp.
34	copy number alteration*.mp.

35	fusion positive.mp.	
36	fusion negative.mp.	
37	"t(11;19)".mp.	
38	CRTC1.mp.	
39	CRTC3.mp.	
40	MAML1.mp.	
41	MAML2.mp.	
42	MECT1.mp.	
43	or/1-4	
44	or/5-42	
45	43 and 44	**3674**
	**Web of Science**	
1	TOPIC: (mec)<br>Indexes=SCI-EXPANDED, SSCI, A&HCI, CPCI-S, CPCI-SSH, BKCI-S, BKCI-SSH, ESCI Timespan=1945-2019	
2	TOPIC: ("mec")<br>Indexes=SCI-EXPANDED, SSCI, A&HCI, CPCI-S, CPCI-SSH, BKCI-S, BKCI-SSH, ESCI Timespan=1945-2019	
3	TOPIC: (mucoepidermoid)<br>Indexes=SCI-EXPANDED, SSCI, A&HCI, CPCI-S, CPCI-SSH, BKCI-S, BKCI-SSH, ESCI Timespan=1945-2019	
4	#2 AND #3<br>Indexes=SCI-EXPANDED, SSCI, A&HCI, CPCI-S, CPCI-SSH, BKCI-S, BKCI-SSH, ESCI Timespan=1945-2019	
5	TOPIC: (adenosquamous)<br>Indexes=SCI-EXPANDED, SSCI, A&HCI, CPCI-S, CPCI-SSH, BKCI-S, BKCI-SSH, ESCI Timespan=1945-2019	
6	TOPIC: (("next generation" or "whole genome" or "whole exome" or nucleotide) near/1 sequenc*)<br>Indexes=SCI-EXPANDED, SSCI, A&HCI, CPCI-S, CPCI-SSH, BKCI-S, BKCI-SSH, ESCI Timespan=1945-2019	
7	TOPIC: ("molecular analys?s")<br>Indexes=SCI-EXPANDED, SSCI, A&HCI, CPCI-S, CPCI-SSH, BKCI-S, BKCI-SSH, ESCI Timespan=1945-2019	
8	TOPIC: ("genetic analys?s")<br>Indexes=SCI-EXPANDED, SSCI, A&HCI, CPCI-S, CPCI-SSH, BKCI-S, BKCI-SSH, ESCI Timespan=1945-2019	
9	TOPIC: ("DNA sequenc*")<br>Indexes=SCI-EXPANDED, SSCI, A&HCI, CPCI-S, CPCI-SSH, BKCI-S, BKCI-SSH, ESCI Timespan=1945-2019	
10	TOPIC: ("chromosome map*")<br>Indexes=SCI-EXPANDED, SSCI, A&HCI, CPCI-S, CPCI-SSH, BKCI-S, BKCI-SSH, ESCI Timespan=1945-2019	

11	TOPIC: ("epigenetic test*")<br>Indexes=SCI-EXPANDED, SSCI, A&HCI, CPCI-S, CPCI-SSH, BKCI-S, BKCI-SSH, ESCI Timespan=1945-2019
12	TOPIC: ("DNA methylation")<br>Indexes=SCI-EXPANDED, SSCI, A&HCI, CPCI-S, CPCI-SSH, BKCI-S, BKCI-SSH, ESCI Timespan=1945-2019
13	TOPIC: ("fluorescence in-situ hybridization")<br>Indexes=SCI-EXPANDED, SSCI, A&HCI, CPCI-S, CPCI-SSH, BKCI-S, BKCI-SSH, ESCI Timespan=1945-2019
14	TOPIC: ("fluorescence in situ hybridization")<br>Indexes=SCI-EXPANDED, SSCI, A&HCI, CPCI-S, CPCI-SSH, BKCI-S, BKCI-SSH, ESCI Timespan=1945-2019
15	TOPIC: ("FISH")<br>Indexes=SCI-EXPANDED, SSCI, A&HCI, CPCI-S, CPCI-SSH, BKCI-S, BKCI-SSH, ESCI Timespan=1945-2019
16	TOPIC: ("polymerase chain reaction")<br>Indexes=SCI-EXPANDED, SSCI, A&HCI, CPCI-S, CPCI-SSH, BKCI-S, BKCI-SSH, ESCI Timespan=1945-2019
17	TOPIC: ("RT-PCR")<br>Indexes=SCI-EXPANDED, SSCI, A&HCI, CPCI-S, CPCI-SSH, BKCI-S, BKCI-SSH, ESCI Timespan=1945-2019
18	TOPIC: ("reverse-transcription PCR")<br>Indexes=SCI-EXPANDED, SSCI, A&HCI, CPCI-S, CPCI-SSH, BKCI-S, BKCI-SSH, ESCI Timespan=1945-2019
19	TOPIC: (karyotyping)<br>Indexes=SCI-EXPANDED, SSCI, A&HCI, CPCI-S, CPCI-SSH, BKCI-S, BKCI-SSH, ESCI Timespan=1945-2019
20	TOPIC: ("genomic profil*")<br>Indexes=SCI-EXPANDED, SSCI, A&HCI, CPCI-S, CPCI-SSH, BKCI-S, BKCI-SSH, ESCI Timespan=1945-2019
21	TOPIC: ("mutation analys?s")<br>Indexes=SCI-EXPANDED, SSCI, A&HCI, CPCI-S, CPCI-SSH, BKCI-S, BKCI-SSH, ESCI Timespan=1945-2019
22	TOPIC: ("somatic mutation*")<br>Indexes=SCI-EXPANDED, SSCI, A&HCI, CPCI-S, CPCI-SSH, BKCI-S, BKCI-SSH, ESCI Timespan=1945-2019
23	TOPIC: ("copy number alteration*")<br>Indexes=SCI-EXPANDED, SSCI, A&HCI, CPCI-S, CPCI-SSH, BKCI-S, BKCI-SSH, ESCI Timespan=1945-2019
24	TOPIC: ("fusion positive")<br>Indexes=SCI-EXPANDED, SSCI, A&HCI, CPCI-S, CPCI-SSH, BKCI-S, BKCI-SSH, ESCI Timespan=1945-2019
25	TOPIC: ("fusion negative")<br>Indexes=SCI-EXPANDED, SSCI, A&HCI, CPCI-S, CPCI-SSH, BKCI-S, BKCI-SSH, ESCI Timespan=1945-2019
26	TOPIC: ("t(11;19)")<br>Indexes=SCI-EXPANDED, SSCI, A&HCI, CPCI-S, CPCI-SSH, BKCI-S, BKCI-SSH, ESCI Timespan=1945-2019
27	TOPIC: (gene near/1 fusion)

	Indexes=SCI-EXPANDED, SSCI, A&HCI, CPCI-S, CPCI-SSH, BKCI-S, BKCI-SSH, ESCI Timespan=1945-2019	
28	TOPIC: (epigenomics)<br>Indexes=SCI-EXPANDED, SSCI, A&HCI, CPCI-S, CPCI-SSH, BKCI-S, BKCI-SSH, ESCI Timespan=1945-2019	
29	TOPIC: (CRTC1)<br>Indexes=SCI-EXPANDED, SSCI, A&HCI, CPCI-S, CPCI-SSH, BKCI-S, BKCI-SSH, ESCI Timespan=1945-2019	
30	TOPIC: (CRTC3)<br>Indexes=SCI-EXPANDED, SSCI, A&HCI, CPCI-S, CPCI-SSH, BKCI-S, BKCI-SSH, ESCI Timespan=1945-2019	
31	TO32PIC: (MAML1)<br>Indexes=SCI-EXPANDED, SSCI, A&HCI, CPCI-S, CPCI-SSH, BKCI-S, BKCI-SSH, ESCI Timespan=1945-2019	
32	TOPIC:34 (MAML2)<br>Indexes=SCI-EXPANDED, SSCI, A&HCI, CPCI-S, CPCI-SSH, BKCI-S, BKCI-SSH, ESCI Timespan=1945-2019	
33	TOPIC: (MECT1)<br>Indexes=SCI-EXPANDED, SSCI, A&HCI, CPCI-S, CPCI-SSH, BKCI-S, BKCI-SSH, ESCI Timespan=1945-2019	
34	#33 OR #32 OR #31 OR #30 OR #29 OR #28 OR #27 OR #26 OR #25 OR #24 OR #23 OR #22 OR #21 OR #20 OR #19 OR #18 OR #17 OR #16 OR #14 OR #13 OR #12 OR #11 OR #10 OR #9 OR #8 OR #7 OR #6<br>Indexes=SCI-EXPANDED, SSCI, A&HCI, CPCI-S, CPCI-SSH, BKCI-S, BKCI-SSH, ESCI Timespan=1945-2019	
35	#34 OR #15<br>Indexes=SCI-EXPANDED, SSCI, A&HCI, CPCI-S, CPCI-SSH, BKCI-S, BKCI-SSH, ESCI Timespan=1945-2019	
36	#5 OR #3<br>Indexes=SCI-EXPANDED, SSCI, A&HCI, CPCI-S, CPCI-SSH, BKCI-S, BKCI-SSH, ESCI Timespan=1945-2019	
37	#36 AND #34<br>Indexes=SCI-EXPANDED, SSCI, A&HCI, CPCI-S, CPCI-SSH, BKCI-S, BKCI-SSH, ESCI Timespan=1945-2019	
38	#36 AND #35<br>Indexes=SCI-EXPANDED, SSCI, A&HCI, CPCI-S, CPCI-SSH, BKCI-S, BKCI-SSH, ESCI Timespan=1945-2019	**627**

S-Table 2: Criteria applied to score the quality appraisal using an adapted version of the Joanna Briggs Institute Critical Appraisal tool for Case Series *

Applied criteria	How to assess items
Were there clear criteria for inclusion in the case series?	The authors should provide clear inclusion (and exclusion criteria where appropriate) for the study participants. The inclusion/exclusion criteria should be specified (e.g., risk, stage of disease progression) with sufficient detail and all the necessary information critical to the study. *Were the types of tumours included accurately described?*
Were valid methods or determinations or technics used for identification of the condition for all participants included in the case series?	Many health problems are not easily diagnosed or defined and some measures may not be capable of including or excluding appropriate levels or stages of the health problem. If the outcomes were assessed based on existing definitions or diagnostic criteria, then the answer to this question is likely to be yes. If the outcomes were assessed using observer reported, or self-reported scales, the risk of over- or under-reporting is increased, and objectivity is compromised. Importantly, determine if the measurement tools used were validated instruments as this has a significant impact on outcome assessment validity. *Were the pathologic features used to diagnose the tumours adequately described? Eg. WHO criteria*
Did the case series have consecutive inclusion of participants?	Studies that indicate a consecutive inclusion are more reliable than those that do not. *Were all cases included between certain dates?*
Did the case series have complete inclusion of participants?	The completeness of a case series contributes to its reliability. Studies that indicate a complete inclusion are more reliable than those that do not. *Were all cases found tested (or only a subset)?*
Was there clear reporting of the demographics of the participants in the study?	The case series should clearly describe relevant participant's demographics such as the following information where relevant: participant's age, sex, education, geographic region, ethnicity, time period, education. *Was at least age and sex reported for each patient in a table?*
Was there clear reporting of clinical information of the participants?	There should be clear reporting of clinical information of the participants such as the following information where relevant: disease status, comorbidities, stage of disease, previous interventions/treatment, results of diagnostic tests, etc.. *Was at least body site, TNM, previous treatment reported for each patient?*
Were the outcomes or follow-up results of cases clearly reported? NA for this study.	*We did not evaluate this question due to the nature of our study which focussed on molecular testing.*

Was the condition/tumour measured in a standard, reliable way for all participants included in the case series?	The study should clearly describe the method of measurement of the condition. This should be done in a standard (i.e. same way for all patients) and reliable (i.e. repeatable and reproducible results) way. *Were the molecular tests performed adequately described?*
Was there clear reporting of the molecular features information?	Certain diseases or conditions vary in prevalence across different geographic regions and populations (e.g. women vs. men, sociodemographic variables between countries). The study sample should be described in sufficient detail so that other researchers can determine if it is comparable to the population of interest to them. *Were the mutations and/or other molecular results described in detail?*
Was statistical analysis appropriate?	As with any consideration of statistical analysis, consideration should be given to whether there was a more appropriate alternate statistical method that could have been used. The methods section of studies should be detailed enough for reviewers to identify which analytical techniques were used and whether these were suitable. *Were simple descriptive statistics, proportion, differences between groups provided?*

*Adapted from https://joannabriggs.org/sites/default/files/2020-08/Checklist_for_Case_Series.pdf

S-Table 6: PRISMA 2020 Checklist for the systematic review "Mucoepidermoid carcinoma (MEC) and adenosquamous carcinoma (ASC): the same or different? A systematic review of molecular pathology to aid in classification."

Section and Topic	Item #	Checklist item	Reported in page
**TITLE**			
Title	1	Identify the report as a systematic review.	1
**ABSTRACT**			
Abstract	2	See the PRISMA 2020 for Abstracts checklist.	2-3
**INTRODUCTION**			
Rationale	3	Describe the rationale for the review in the context of existing knowledge.	4-7
Objectives	4	Provide an explicit statement of the objective(s) or question(s) the review addresses.	7
**METHODS**			
Eligibility criteria	5	Specify the inclusion and exclusion criteria for the review and how studies were grouped for the syntheses.	8-9
Information sources	6	Specify all databases, registers, websites, organisations, reference lists and other sources searched or consulted to identify studies. Specify the date when each source was last searched or consulted.	7-8
Search strategy	7	Present the full search strategies for all databases, registers and websites, including any filters and limits used.	Supplementary materials
Selection process	8	Specify the methods used to decide whether a study met the inclusion criteria of the review, including how many reviewers screened each record and each report retrieved, whether they worked independently, and if applicable, details of automation tools used in the process.	9
Data collection process	9	Specify the methods used to collect data from reports, including how many reviewers collected data from each report, whether they worked independently, any processes for obtaining or confirming data from study investigators, and if applicable, details of automation tools used in the process.	9-10
Data items	10a	List and define all outcomes for which data were sought. Specify whether all results that were compatible with each outcome domain in each study were sought (e.g. for all measures, time points, analyses), and if not, the methods used to decide which results to collect.	10
	10b	List and define all other variables for which data were sought (e.g. participant and intervention characteristics, funding sources). Describe any assumptions made about any missing or unclear information.	10
Study risk of bias assessment	11	Specify the methods used to assess risk of bias in the included studies, including details of the tool(s) used, how many reviewers assessed each study and whether they worked independently, and if applicable, details of automation tools used in the process.	10 + Supplementary materials
Effect measures	12	Specify for each outcome the effect measure(s) (e.g. risk ratio, mean difference) used in the synthesis or presentation of results.	Not applicable
Synthesis methods	13a	Describe the processes used to decide which studies were eligible for each synthesis (e.g. tabulating the study intervention characteristics and comparing against the planned groups for each synthesis (item #5)).	10

Section and Topic	Item #	Checklist item	Reported in page
	13b	Describe any methods required to prepare the data for presentation or synthesis, such as handling of missing summary statistics, or data conversions.	Not applicable
	13c	Describe any methods used to tabulate or visually display results of individual studies and syntheses.	Not applicable
	13d	Describe any methods used to synthesize results and provide a rationale for the choice(s). If meta-analysis was performed, describe the model(s), method(s) to identify the presence and extent of statistical heterogeneity, and software package(s) used.	10
	13e	Describe any methods used to explore possible causes of heterogeneity among study results (e.g. subgroup analysis, meta-regression).	Not applicable
	13f	Describe any sensitivity analyses conducted to assess robustness of the synthesized results.	Not applicable
Reporting bias assessment	14	Describe any methods used to assess risk of bias due to missing results in a synthesis (arising from reporting biases).	Not applicable
Certainty assessment	15	Describe any methods used to assess certainty (or confidence) in the body of evidence for an outcome.	Not applicable
**RESULTS**			
Study selection	16a	Describe the results of the search and selection process, from the number of records identified in the search to the number of studies included in the review, ideally using a flow diagram.	11+<br>Figure 1
	16b	Cite studies that might appear to meet the inclusion criteria, but which were excluded, and explain why they were excluded.	Figure 1
Study characteristics	17	Cite each included study and present its characteristics.	Table 1 + Supplementary materials
Risk of bias in studies	18	Present assessments of risk of bias for each included study.	Supplementary materials
Results of individual studies	19	For all outcomes, present, for each study: (a) summary statistics for each group (where appropriate) and (b) an effect estimate and its precision (e.g. confidence/credible interval), ideally using structured tables or plots.	11-16<br>+Table 1<br>+Supplementary materials
Results of syntheses	20a	For each synthesis, briefly summarise the characteristics and risk of bias among contributing studies.	11-16
	20b	Present results of all statistical syntheses conducted. If meta-analysis was done, present for each the summary estimate and its precision (e.g. confidence/credible interval) and measures of statistical heterogeneity. If comparing groups, describe the direction of the effect.	Not applicable
	20c	Present results of all investigations of possible causes of heterogeneity among study results.	Not applicable
	20d	Present results of all sensitivity analyses conducted to assess the robustness of the synthesized results.	Not applicable

Section and Topic	Item #	Checklist item	Reported in page
Reporting biases	21	Present assessments of risk of bias due to missing results (arising from reporting biases) for each synthesis assessed.	Not applicable
Certainty of evidence	22	Present assessments of certainty (or confidence) in the body of evidence for each outcome assessed.	Not applicable
**DISCUSSION**			
Discussion	23a	Provide a general interpretation of the results in the context of other evidence.	16-18
	23b	Discuss any limitations of the evidence included in the review.	17-20
	23c	Discuss any limitations of the review processes used.	18
	23d	Discuss implications of the results for practice, policy, and future research.	19-22<br>Table 3
**OTHER INFORMATION**			
Registration and protocol	24a	Provide registration information for the review, including register name and registration number, or state that the review was not registered.	7
	24b	Indicate where the review protocol can be accessed, or state that a protocol was not prepared.	7
	24c	Describe and explain any amendments to information provided at registration or in the protocol.	Not applicable
Support	25	Describe sources of financial or non-financial support for the review, and the role of the funders or sponsors in the review.	24
Competing interests	26	Declare any competing interests of review authors.	24
Availability of data, code and other materials	27	Report which of the following are publicly available and where they can be found: template data collection forms; data extracted from included studies; data used for all analyses; analytic code; any other materials used in the review.	Not applicable
